# Case Report: Coexistence of advanced systemic mastocytosis, chronic myelomonocytic Leukemia, and t(11;14)-positive plasma-cell neoplasm: a rare triple-clonal hematologic case

**DOI:** 10.3389/fonc.2026.1982665

**Published:** 2026-10-19

**Authors:** Jun Yin, Jingyuan Luo, Linlin Huang, Ling Wang, Mingwei Zhong, Maoqin Li, Shihua Huang

**Affiliations:** 1 Department of Hematology, Second People’s Hospital of Yibin City, Sichuan, China; 2 Department of Pathology, Sichuan Kingmed Center for Clinical Laboratory, Sichuan, China; 3 Hematopathy Diagnosis Center, Sichuan Kingmed Center for Clinical Laboratory, Sichuan, China

**Keywords:** chronic myelomonocytic leukemia, clonal hematopoiesis, KIT D816V, plasma-cell neoplasm, SM-AHN, systemic mastocytosis, T(11;14)

## Abstract

**Background:**

SM-AHN usually coexists with myeloid disorders, whereas plasma-cell neoplasms are rare. We report a case of advanced systemic mastocytosis, CMML, and a t(11;14)-positive monoclonal plasma-cell disorder, highlighting the diagnostic challenge of overlapping clinical, biochemical, and morphologic features.

**Case presentation:**

A middle-aged man presented with months of watery diarrhea, recurrent abdominal discomfort, weight loss, markedly elevated alkaline phosphatase, and persistent monocytosis. Bone marrow examination showed dense aggregates of atypical mast cells involving about 50% of marrow cellularity, with aberrant CD25 expression, and KIT D816V (variant allele frequency, 12.10%), supporting advanced systemic mastocytosis. Sustained absolute monocytosis, classical-monocyte predominance up to 98%, and mutations in *TET2*, *SRSF2*, and *RUNX1* supported concurrent CMML-1. Marrow plasma-cell infiltration, markedly elevated lambda free light chains, a low κ/λ ratio, and t(11;14) identified a third clonal process consistent with an IgG lambda plasma-cell neoplasm. Together, these findings established coexistence of advanced SM, CMML, and a plasma-cell disorder, with early symptomatic and biochemical improvement after treatment targeting the mast-cell and myeloid components.

**Conclusion:**

This case highlights the value of integrating morphology, flow cytometry, cytogenetics, and next-generation sequencing in complex hematologic presentations. The coexistence of SM, CMML, and a t(11;14)-positive plasma-cell neoplasm is exceptionally rare, with important implications for diagnosis, risk assessment, and treatment.

## 1 Introduction

Systemic mastocytosis (SM) is a clonal disorder arising from abnormal proliferation and accumulation of neoplastic mast cells in the bone marrow and various extracutaneous organs. Although considered a rare hematologic disease, recent population-level studies indicate that its prevalence is higher than previously assumed, with most adult cases falling under systemic rather than cutaneous variants. The fifth edition of the World Health Organization Classification of Haematolymphoid Tumours (WHO-HAEM5) classifies systemic mastocytosis into bone marrow mastocytosis, indolent systemic mastocytosis, smoldering systemic mastocytosis, aggressive systemic mastocytosis, systemic mastocytosis with an associated hematologic neoplasm (SM-AHN), and mast cell leukemia.

Advances in molecular diagnostics have substantially reshaped the understanding of SM pathogenesis. More than 90% of adult SM patients carry the activating KIT D816V mutation, which confers constitutive signaling through the KIT receptor and supports mast cell survival and expansion. However, in many patients, especially those with SM-AHN, additional somatic mutations occur in genes regulating epigenetic modification and RNA splicing, such as *TET2*, *SRSF2*, *ASXL1*, and *RUNX1* mirroring the mutational pattern seen in other myeloid neoplasms (1). These findings support the concept that SM-AHN typically originates from an early hematopoietic progenitor with multilineage potential rather than from a mast cell-restricted precursor. The presence of *KIT* D816V across mast cells, monocytes, and granulocytes in many SM-AHN patients further reinforces the hypothesis of a common ancestral clone shared between SM and the associated myeloid malignancy (2).

SM-AHN is not a rare entity; studies of large patient cohorts show that approximately 30-40% of individuals with SM harbor a concurrent hematologic neoplasm. In a landmark series of 342 patients, nearly half had a myeloid neoplasm as their AHN component, with chronic myelomonocytic leukemia (CMML), myelodysplastic syndromes (MDS), and myeloproliferative neoplasms being the most frequent associations (3). More detailed analyses of SM-AHN cohorts revealed that CMML accounts for roughly 25-30% of all AHN cases and presents with characteristic molecular alterations, most notably in *TET2* and *SRSF2* (4). These shared mutations form the biological foundation for recent models of SM-CMML clonal evolution. Rechsteiner et al. elegantly demonstrated that SM-CMML can arise from a genetically diverse founding clone carrying *KIT*, *TET2*, and *RUNX1* mutations, which subsequently evolves into divergent mast cell-dominant and monocyte-dominant subclones. This model provides a mechanistic explanation for why SM-CMML often presents with extensive organ infiltration, elevated mast-cell burden, and progressive monocytosis.

CMML itself is classified as a myelodysplastic/myeloproliferative neoplasm characterized by persistent monocytosis, dysplastic morphology, and recurrent somatic mutations involving RNA splicing and epigenetic regulators. The 2024 update by Patnaik and Tefferi highlights that *TET2*, *SRSF2*, and *RUNX1* mutations often coexist in CMML and carry prognostic significance (5). When CMML occurs as part of SM-AHN, the disease course is frequently more aggressive, with higher rates of splenomegaly, constitutional symptoms, and organ infiltration than in isolated CMML. Comparative studies show that SM-CMML patients also have different molecular features, including higher rates of KIT D816V and CBL mutations, as well as inferior survival (6).

Unlike the well-established association between SM and myeloid neoplasms, the coexistence of SM and plasma-cell diseases, such as monoclonal gammopathy or multiple myeloma (MM) is distinctly uncommon. Only isolated case reports have documented SM with plasma-cell dyscrasias, including cases associated with smoldering MM (PMID: 27293930, PMID: 19147227, PMID: 18061667). Biologically, mast cells may promote plasma-cell survival by secreting cytokines such as IL-6 and stem cell factor (SCF), which are recognized growth and differentiation factors in MM. IL-6 was demonstrated decades ago to support myeloma proliferation via paracrine signaling, and later studies showed that SCF can further enhance IL-6-driven myeloma cell growth (7). Additionally, mast cells can promote lymphoplasmacytic proliferation through CD40-CD154 interactions, as shown in Waldenström macroglobulinemia models. These mechanistic links explain why plasma-cell dyscrasias may rarely emerge alongside SM, though they typically develop from an entirely independent B-cell lineage rather than sharing the KIT-driven origin of SM.

Despite these mechanistic insights, simultaneous coexistence of SM, CMML, and a clonal plasma-cell neoplasm in a single patient has rarely been described in the available literature. Existing reports describe SM associated with one additional clonal hematologic neoplasm, commonly CMML, rarely MM, but not with both diseases concurrently. The emergence of three distinct hematologic malignancies in the same individual raises important clinical and biological questions, including the timeline of clonal evolution, the interaction of mast cell-driven inflammation with myeloid and plasma-cell proliferation, and the impact of divergent mutational pathways on disease progression and therapeutic decision-making.

In this context, the present report describes a patient who presented with persistent gastrointestinal symptoms and cytopenias and was ultimately diagnosed with advanced systemic mastocytosis, CMML-1, and a t(11;14)-positive plasma-cell neoplasm. To our knowledge, no similar case has been previously reported. This unique constellation of diagnoses underscores the necessity of comprehensive diagnostic assessment, integrating histopathology, flow cytometry, molecular genetics, and clinical findings, when a patient’s symptoms cannot be fully explained by a single hematologic condition.

## 2 Case presentation

A 65-year-old man with no known chronic illnesses presented with a four-month history of persistent watery diarrhea. Initial evaluation at a local hospital revealed chronic non-atrophic gastritis and duodenitis, but his symptoms did not improve with standard therapy. Laboratory testing during this period showed leukocytes of 5.61 ×10^9^/L, an absolute monocytosis of 1.54 ×10^9^/L, hemoglobin 147 g/L, and thrombocytopenia (66 ×10^9^/L), prompting referral to our institution for further workup.

On admission, physical examination revealed splenomegaly and bilateral lower-extremity edema. Serial blood counts confirmed sustained absolute monocytosis, increasing from 1.54 ×10^9^/L to 1.93 ×10^9^/L, together with normocytic anemia (100 g/L) and persistent thrombocytopenia (62 ×10^9^/L). Biochemical studies were notable for a markedly elevated alkaline phosphatase level (1,145.3 U/L) and ferritin > 1,500 ng/mL, whereas renal and hepatic indices remained within normal limits.

Contrast-enhanced CT demonstrated marked splenomegaly, with a craniocaudal diameter of approximately 13 cm, accompanied by multiple mildly enlarged retroperitoneal lymph nodes. ^18F-FDG PET-CT showed intense gastric wall uptake (SUV_max 19.1) with diffusely increased splenic metabolism and mildly hypermetabolic mediastinal and abdominal lymph nodes, raising suspicion for an infiltrative hematologic process. Endoscopic reevaluation with biopsies from the gastric antrum, body, and duodenum revealed chronic active gastritis, duodenal ulcers, and mucosal inflammation, without specific infiltrative lesions (**Figure 1**).

**Figure 1:**
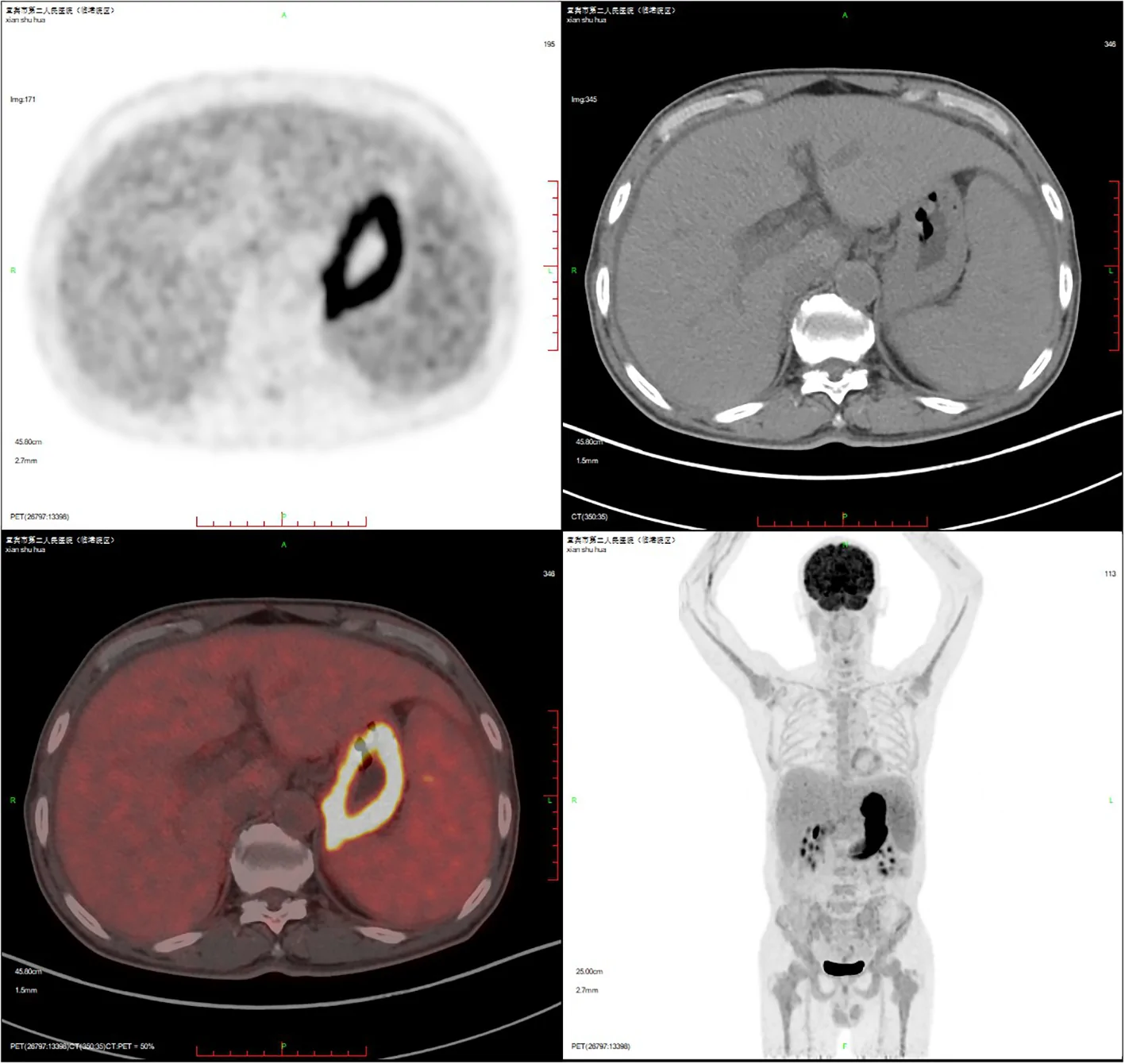
FDG-PET/CT demonstrates thickening of gastric wall and hyperintense FDG uptake. **(A)** Axial PET image showing intense FDG uptake in the thickened gastric wall. **(B)** Axial CT image showing marked splenomegaly. **(C)** Axial fused PET/CT image demonstrating intense FDG uptake in the gastric wall. **(D)** Maximum-intensity-projection PET image showing diffusely increased splenic uptake and mildly increased uptake in multiple abdominal lymph nodes.

Given the persistent diarrhea, cytopenias, splenomegaly, and progressive monocytosis, bone marrow aspiration and trephine biopsy were performed. Aspirate smears were examined using Wright-Giemsa and toluidine blue staining. Following morphologic reassessment of the bone-marrow aspirate, atypical mast cells accounted for 18.5% of nucleated cells. Despite the extensive mast-cell infiltration observed on bone-marrow biopsy, the mast-cell proportion in the bone-marrow aspirate was 18.5%. Current WHO-HAEM5 criteria define mast cell leukemia by ≥20% mast cells in the bone-marrow aspirate smear. Because this threshold was not met, the diffuse biopsy infiltration alone was insufficient to establish mast cell leukemia, supporting classification as advanced systemic mastocytosis with an associated hematologic neoplasm. Immunohistochemistry of the bone marrow biopsy showed CD117^-^, CD25^-^, and tryptase-positive mast cells together with CD138-positive plasma cells. Multiparameter flow cytometry of the bone marrow aspirate identified a mast-cell population (12.33%) with bright CD117 and aberrant CD25 expression and a monoclonal lambda-restricted plasma-cell population (3.37%). Monocyte repartitioning showed 98% classical CD14^+^/CD16^−^ monocytes, supporting a clonal myeloid process (**Figure 2**; **Supplementary Table 1**).

**Figure 2:**
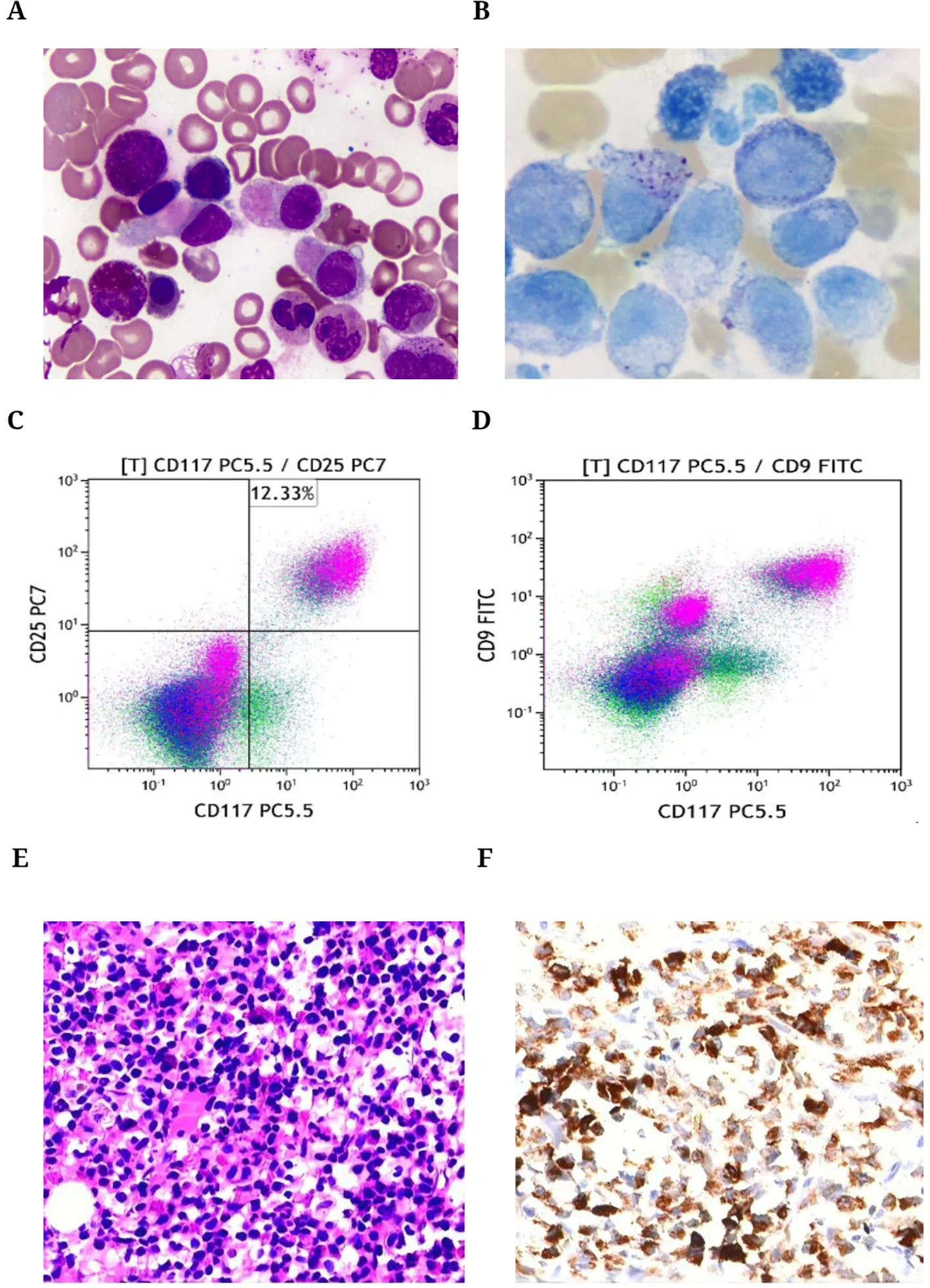
Representative morphology and immunophenotypic features of SM in the BM. **(A, B)** Wright-Giemsa and toluidine blue staining of bone marrow × 1000 shows abnormal mast cells with positive toluidine blue staining. **(C, D)** Flow cytometry of the BM aspirate detected a mast cell population with immunophenotypic abnormalities,whose immunostains were positive for CD117, CD25, CD9. **(E, F)** HE × 200, IHC × 400 shows mast cells Tryptase positivity in the BM biopsy.

A myeloid-malignancy-focused NGS panel performed on bone marrow aspirate DNA identified mutations in *TET2*, *SRSF2*, *RUNX1*, and *KIT D816V*, together with a *TET2* exons 3–11 deletion. These findings established advanced systemic mastocytosis with multilineage involvement. Concurrently, serum free-light-chain analysis showed a markedly elevated lambda free light-chain level of 2,274.69 mg/L, with a free kappa level of 28.98 mg/L and a markedly suppressed κ/λ ratio of 0.0127. Serum and urine immunofixation electrophoresis demonstrated a monoclonal lambda light-chain component. Bone-marrow biopsy showed ≥ 20% CD138-positive plasma-cell infiltration, and CD138-enriched FISH identified t(11;14)(q13;q32)/IGH::CCND1, supporting the presence of a concurrent clonal plasma-cell neoplasm (**Figure 3**). Peripheral-blood smears showed increased circulating monocytes with abundant gray-blue cytoplasm and irregular, folded nuclei. Given the sustained absolute monocytosis (1.54-1.93 ×10^9^/L for > 3 months), classical-monocyte predominance, and the presence of *TET2*, *SRSF2*, and *RUNX1* mutations in peripheral blood, the patient simultaneously met WHO/ICC criteria for CMML-1, proliferative subtype (**Figure 4**).

**Figure 3:**
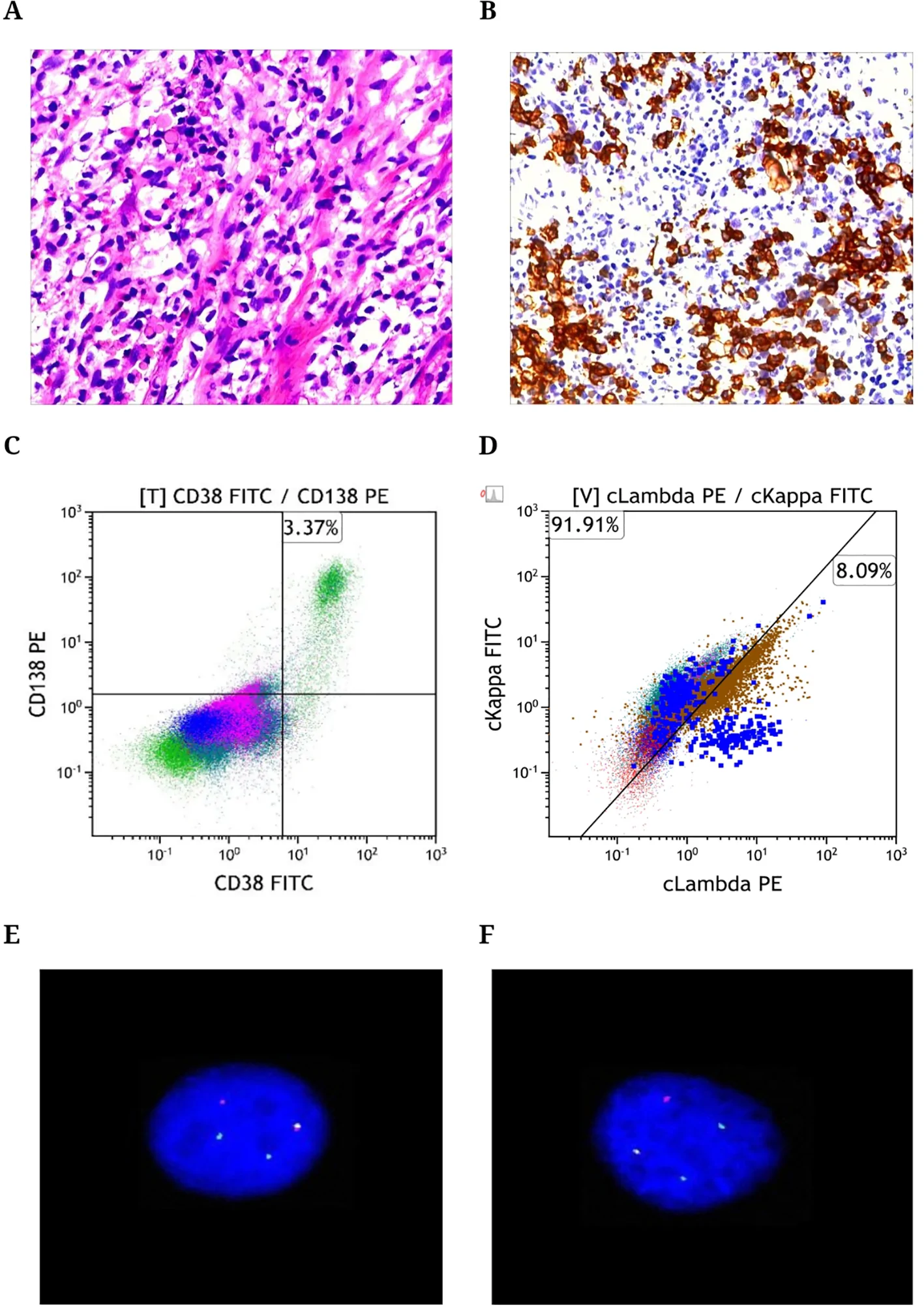
Representative morphologic, immunophenotypic, and FISH features of the plasma-cell neoplasm in the bone marrow. **(A, B)** HE × 400,IHC × 400 shows mature plasma cells CD138 positivity in the BM biopsy. **(C, D)** Flow cytometric analysis of the bone-marrow aspirate showing the plasma-cell population and intracellular kappa/lambda light-chain distribution. **(E, F)** FISH × 1000 demonstrates t(11;14)/IGH::CCND1 fusion in the clonal plasma-cell population.

**Figure 4:**
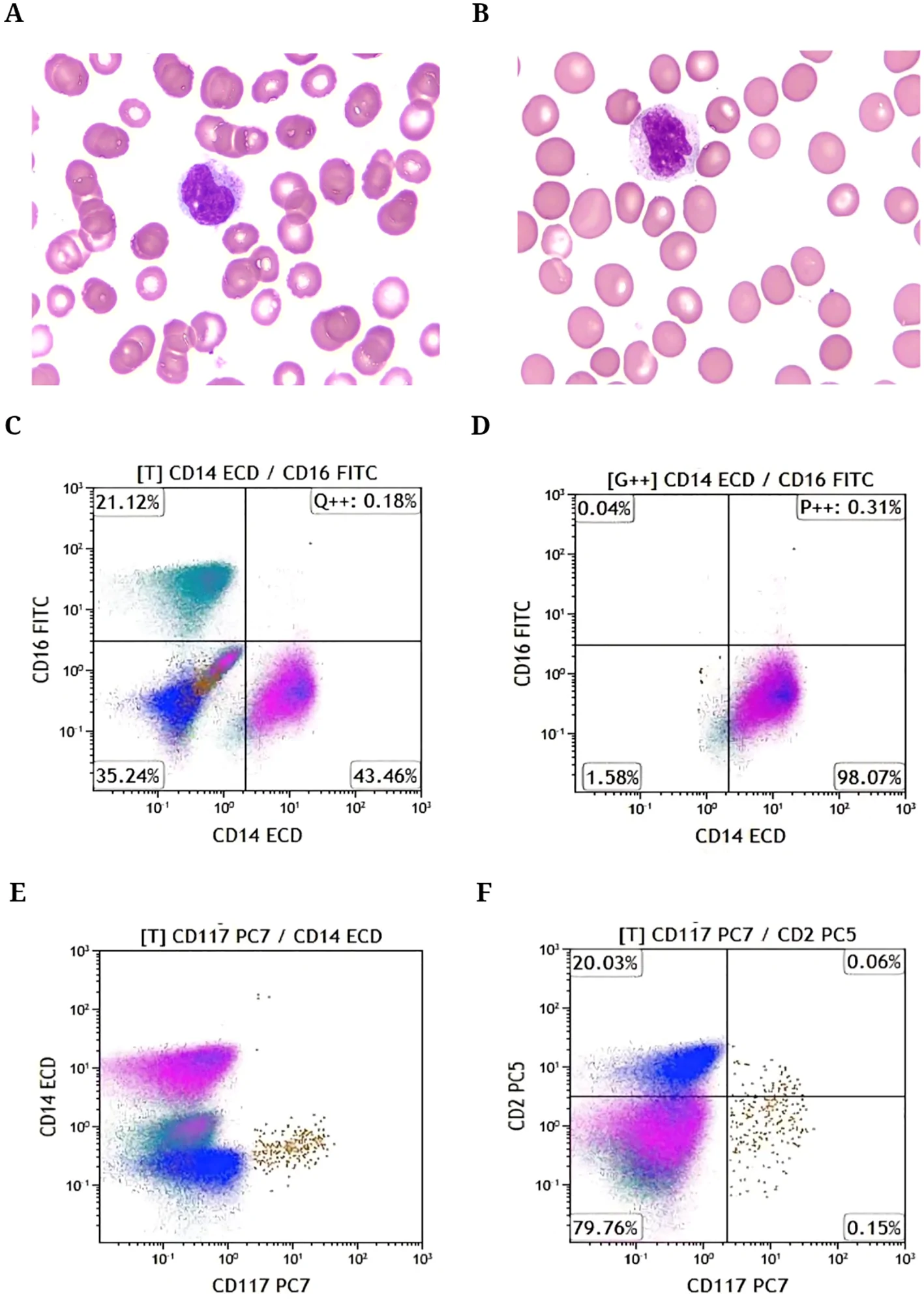
Representative morphology and immunophenotypic features of CMML in the PB. **(A, B)** Wright-Giemsa staining × 1000 shows circulating monocytes with abundant gray-blue cytoplasm and irregular, folded nuclei in the peripheral blood. **(C, D)** Flow cytometry of peripheral blood shows marked predominance of classical CD14+/CD16− monocytes. **(E, F)** Flow cytometry of peripheral blood identifies a CD117-positive mast-cell population with partial CD2 expression and absent CD14 expression.

Based on the clinical, morphologic, immunophenotypic, molecular, and cytogenetic findings, the patient was diagnosed with advanced systemic mastocytosis associated with CMML-1 and a t(11;14)-positive plasma-cell neoplasm. No unequivocal myeloma-defining event attributable to the plasma-cell clone was documented. Treatment with avapritinib and azacitidine was initiated. After three cycles, the frequency of watery diarrhea decreased, hemoglobin and platelet counts improved, the absolute monocyte count declined, and the alkaline phosphatase level showed a downward trend.

## 3 Results

### 3.1 Clinical presentation and initial laboratory evaluation

The patient presented with a four-month history of persistent watery diarrhea accompanied by progressive fatigue and lower-extremity edema. On admission, physical examination demonstrated splenomegaly. Serial laboratory tests revealed persistent cytopenias characterized by normocytic anemia and thrombocytopenia. The white blood cell count was 6.81 × 10^9^/L (reference range, 3.5-9.5 × 10^9^/L), while the absolute monocyte count remained elevated, ranging from 1.54 × 10^9^/L to 1.93 × 10^9^/L (reference range, < 0.5 × 10^9^/L) over multiple measurements, fulfilling the requirement for sustained monocytosis. Hemoglobin decreased from 147 g/L at the outside hospital to 100 g/L at presentation (reference range, 130–175 g/L), and the platelet count was 62 × 10^9^/L (reference range, 125-350 × 10^9^/L). Biochemical evaluation showed an alkaline phosphatase level of 1,145.3 U/L (reference range, 45–125 U/L) and serum ferritin > 1,500 ng/mL (reference range, 30–300 ng/mL), while liver and renal function were otherwise unremarkable. Serum lambda free light chain was markedly elevated at 2,274.69 mg/L (reference range, 5.71-26.3 mg/L), with a κ/λ ratio of 0.0127 (reference range, 0.26-1.65). Serum and urine immunofixation electrophoresis confirmed monoclonal lambda light chains. These findings indicated the coexistence of a clonal myeloid process and a monoclonal plasma-cell disorder.

### 3.2 Imaging findings

Contrast-enhanced abdominal CT demonstrated marked splenomegaly, with a craniocaudal diameter of approximately 13 cm, accompanied by scattered, mildly enlarged retroperitoneal lymph nodes. No focal hepatic lesions or biliary abnormalities were observed. ^18F-FDG PET-CT revealed irregular thickening of the gastric wall with markedly increased metabolic activity, with a maximum standardized uptake value (SUV_max) of 19.1. Diffusely increased uptake was also noted in the spleen, and multiple small mediastinal and abdominal lymph nodes showed mild hypermetabolism. Bone marrow uptake was slightly increased, without focal osseous lesions or other PET/CT findings suggestive of myeloma-related bone disease. Bone MRI was not performed.

### 3.3 Bone marrow morphology and immunophenotype

Reassessment of the bone-marrow aspirate confirmed that atypical mast cells accounted for 18.5% of nucleated cells, remaining below the 20% threshold for mast cell leukemia. Although the bone-marrow biopsy demonstrated extensive multifocal mast-cell infiltration involving approximately 50% of marrow cellularity, this finding did not supersede the aspirate-based criterion in this case because an adequate aspirate was available. The atypical mast cells showed variable cell size, irregular nuclear contours, abnormal cytoplasmic granulation, and occasional spindle-shaped forms. Plasma cells constituted 5.5% of marrow cells and showed mild atypia. The bone marrow biopsy demonstrated dense, multifocal infiltrates of mast cells involving approximately 50% of the marrow cellularity, accompanied by an MF-2 to MF-3 grade reticulin fibrosis pattern. Immunohistochemical staining confirmed strong expression of CD117 and tryptase in the mast cell population, along with aberrant CD25 positivity. Plasma cells were highlighted by CD138 staining and demonstrated lambda light-chain restriction consistent with monoclonal differentiation.

Flow cytometry further supported these findings, identifying an expanded mast cell population representing 12.33% of total events, characterized by bright CD117 expression with co-expression of CD25, confirming aberrant mast cell immunophenotype. The plasma cell fraction comprised 3.37% of total nucleated cells and showed cytoplasmic lambda light-chain restriction, aligning with the biochemical findings. Monocyte immunophenotyping demonstrated a markedly skewed distribution, with 98% of monocytes belonging to the classical subset, an immunophenotypic pattern strongly associated with chronic myelomonocytic leukemia. The atypical mast cells showed variable cell size, irregular nuclear contours, abnormal cytoplasmic granulation, and occasional spindle-shaped forms (**Figure 5**).

**Figure 5:**
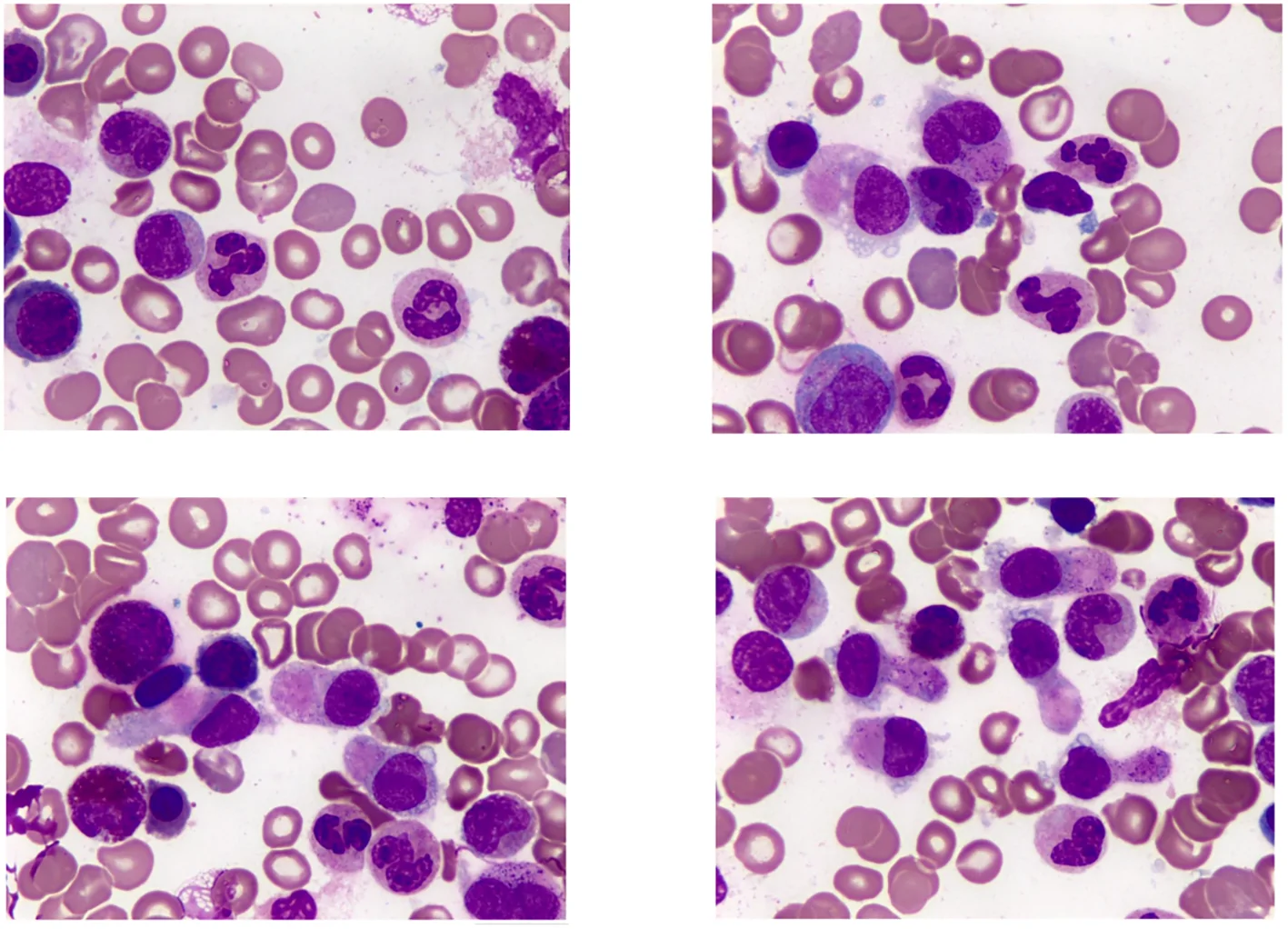
Representative high-power fields of the bone-marrow aspirate showing atypical mast cells. The mast cells show variable cell size, irregular nuclear contours, and abnormal cytoplasmic granulation. These fields were selected to illustrate mast-cell morphology and do not represent a quantitative estimate of the overall mast-cell percentage. Morphologic reassessment of the complete bone-marrow aspirate showed that mast cells accounted for 18.5% of nucleated cells.

### 3.4 Molecular and cytogenetic findings

The dominant lesions included a frameshift mutation in TET2 (p.Pro419fs, VAF 64.9%), consistent with a founding epigenetic clone, and a pathogenic SRSF2 p.Pro95Arg (VAF 44.6%) splice-factor mutation, a hallmark alteration strongly associated with chronic myelomonocytic leukemia. Two independent RUNX1 mutations were detected: a frameshift variant RUNX1 p.Ala338fs (VAF 37.1%) and a nonsense variant RUNX1 p.Arg201Ter (VAF 2.7%), suggesting secondary clonal evolution. A pathogenic KIT p.Asp816Val mutation was present with a VAF of 12.1%, confirming the molecular basis for systemic mastocytosis. Additional mutations included CEBPA p.Glu148Ter (VAF 12.6%) and a missense TET2 p.Arg1359Cys variant (VAF 7.1%). Importantly, copy-number analysis identified a deletion encompassing exons 3–11 of TET2, providing further evidence of biallelic TET2 inactivation.

Cytogenetic assessment by fluorescence *in situ* hybridization revealed a t(11;14)(q13;q32) translocation, consistent with a cyclin-D1/IGH rearrangement characteristic of plasma-cell neoplasms. Other tested plasma-cell-associated cytogenetic abnormalities, including del(17p), 1q gain, t(4;14), and t(14;16), were not detected (**Table 1**). A dedicated myeloma NGS panel was not performed; therefore, contemporary genomic risk classification according to the IMS/IMWG criteria could not be assigned.

**Table 1:** Molecular and cytogenetic findings.

Gene	Variant	VAF %
TET2	p.Pro419fs	64.9
SRSF2	p.Pro95Arg	44.6
RUNX1	p.Ala338fs	37.1
KIT	p.Asp816Val	12.1
CEBPA	p.Glu148Ter	12.6
TET2	p.Arg1359Cys	7.1
RUNX1	p.Arg201Ter	2.7
TET2 CNV	Exons 3–11 deletion	–

### 3.5 Integrated diagnosis and treatment

The integration of clinical presentation, imaging findings, bone marrow morphology, immunophenotype, and molecular profiling supported a diagnosis of advanced systemic mastocytosis (AdvSM) associated with a hematologic neoplasm (SM-AHN). The AHN component consisted of CMML-1, evidenced by persistent absolute monocytosis between 1.54-1.93 ×10^9^/L, classical-monocyte predominance of 98%, and co-occurring *TET2*, *SRSF2*, and RUNX1 mutations. In addition, the presence of a markedly elevated serum lambda free light chain concentration of 2,274.69 mg/L, the markedly elevated serum lambda free light-chain concentration of 2,274.69 mg/L, a profoundly suppressed κ/λ ratio of 0.0127, bone-marrow plasma-cell infiltration of 5.5% on aspirate and ≥ 20% on biopsy, and a t(11;14) rearrangement supported the presence of a concurrent t(11;14)-positive plasma-cell neoplasm. No unequivocal myeloma-defining event attributable to the plasma-cell clone was documented. The involved/uninvolved free light-chain ratio was approximately 78.5 and therefore exceeded the > 20 threshold of the 20/20/20 risk model. However, a formal 20/20/20 risk category could not be assigned because a quantitative serum M-protein concentration was unavailable and the biopsy plasma-cell burden was reported as ≥ 20% without a more precise value above the 20% cutoff. Myeloma-directed therapy was therefore not initiated at this stage, and close surveillance of the plasma-cell component was planned while treatment was prioritized toward the clinically active advanced systemic mastocytosis and CMML components.

The patient was initiated on combined therapy with avapritinib, targeting *KIT* D816V-driven mast cell disease, together with hypomethylating therapy directed toward the underlying CMML clone. Avapritinib was selected because KIT D816V is resistant to imatinib, whereas avapritinib has potent inhibitory activity against KIT D816V and is an established treatment option for advanced systemic mastocytosis. Over the subsequent treatment cycles, the patient’s gastrointestinal symptoms notably improved, and the frequency of watery diarrhea decreased substantially. Hematologic parameters demonstrated gradual stabilization, with improvement in hemoglobin levels and platelet counts, and a reduction in absolute monocyte count, reflecting hematologic response within the myeloid compartment. Biochemical abnormalities associated with mast-cell activation, most prominently the markedly elevated alkaline phosphatase level of 1,145.3 U/L, also showed a downward trend. Serial serum free light-chain measurements and repeat immunofixation electrophoresis were not available during the current follow-up period; therefore, the longitudinal response of the plasma-cell component could not be formally assessed.

Given the overlapping hematologic toxicities of azacitidine and avapritinib, particular attention was paid to cytopenias during treatment. Azacitidine is associated with myelosuppression, including anemia, neutropenia, and thrombocytopenia, while avapritinib may also cause thrombocytopenia and carries risks of bleeding and cognitive adverse effects. Accordingly, serial blood counts and clinical signs of bleeding, infection, and neurologic toxicity were monitored during therapy.

## 4 Discussion

This case illustrates an uncommon but clinically meaningful coexistence of three clonal hematologic processes in a single patient: advanced systemic mastocytosis, chronic myelomonocytic leukemia, and a monoclonal plasma-cell disorder harboring t(11;14) (8–11). The diagnostic complexity was compounded by nonspecific gastrointestinal manifestations, striking biochemical abnormalities, and a broad spectrum of peripheral-blood and bone-marrow findings. The constellation of watery diarrhea, recurrent abdominal discomfort, elevated alkaline phosphatase, and persistent monocytosis initially suggested a mixed infiltrative and myeloproliferative process rather than a single disease entity (12, 13). Only after integrating morphology, flow cytometry, cytogenetics, and next-generation sequencing data was the full spectrum of the patient’s disease recognized.

A dominant feature of this patient’s presentation was the mast-cell infiltration demonstrated in both aspirate and biopsy specimens. The presence of atypical mast cells comprising 18.5% of nucleated cells on aspirate, approximately 50% involvement on biopsy, and expression of CD117 and CD25 established the diagnosis of systemic mastocytosis (14, 15). The markedly elevated alkaline phosphatase level (1,145.3 U/L) was concordant with mast-cell-related hepatic and skeletal involvement, a biochemical hallmark frequently associated with advanced disease (16). The molecular detection of KIT D816V with a variant allele frequency of 12.10% provided further confirmation of a pathogenic mast-cell clone and served as a foundation for targeted therapy selection (17).

The second major disease process in this patient was a concurrent myeloid neoplasm consistent with CMML-1, supported by sustained absolute monocytosis ranging from 1.54-1.93 ×10^9^/L, classical-monocyte dominance reaching 98%, and a mutational profile involving *TET2*, *SRSF2*, and *RUNX1*, a triad frequently associated with CMML pathogenesis and disease progression (5, 18, 19). The coexistence of SM and CMML is well described within the SM-AHN category and often dictates a more aggressive clinical trajectory than either disease alone (6, 8, 9). In this patient, the myeloid clone appeared biologically active, as reflected by cytopenias, monocytosis, and the spectrum of mutations associated with aberrant hematopoiesis.

A third clonal component, an IgG lambda plasma-cell disorder with t(11;14) rearrangement added further complexity (20, 21). Although the patient had ≥ 20% clonal plasma cells on bone-marrow biopsy and marked lambda free-light-chain elevation, the involved/uninvolved free-light-chain ratio was below 100, plasma cells were below 60%, and PET/CT showed no focal osseous lesions. Bone MRI was not performed. We therefore did not identify a documented myeloma-defining event and classified this component conservatively as a t(11;14)-positive plasma-cell neoplasm. The involved/uninvolved free-light-chain ratio was approximately 78.5, exceeding the > 20 threshold of the 20/20/20 model. However, the serum M-protein concentration was not quantitatively available, and the biopsy plasma-cell percentage was reported as ≥ 20% without a precise value above the > 20% cutoff. Formal 20/20/20 risk stratification was therefore not possible. The plasma-cell infiltration of 5.5% on aspirate and ≥ 20% on biopsy, together with the profoundly elevated lambda free light chain concentration (2,274.69 mg/L) and suppressed κ/λ ratio (0.0127), met criteria for a coexisting monoclonal plasma-cell neoplasm. Although plasma-cell disorders are occasionally encountered in SM-AHN, their coexistence with both SM and CMML in the same patient is distinctly uncommon. The presence of t(11;14) may have therapeutic relevance if the plasma-cell neoplasm progresses to treatment-requiring disease. Venetoclax has demonstrated activity in t(11;14)-positive multiple myeloma, but it is not established as routine therapy for an untreated asymptomatic plasma-cell disorder. Because no myeloma-defining event was documented and the immediate treatment priority was the clinically active mast-cell and myeloid disease, venetoclax was not added during the reported treatment period (22–24).

The decision to initiate avapritinib in combination with hypomethylating therapy reflected a need to target both the *KIT* D816V-driven mast-cell clone and the molecularly defined myeloid neoplasm. Avapritinib was selected because the patient harbored KIT D816V, a mutation that is generally resistant to imatinib. In contrast, avapritinib directly targets KIT D816V and has demonstrated clinical activity in advanced systemic mastocytosis, including reductions in mast-cell burden and disease-related symptoms. Hypomethylating therapy was selected to address CMML-associated dysplasia and the *TET2*/*SRSF2*/*RUNX1* mutation cluster, which portends a risk of disease evolution if left untreated.

This case underscores the necessity of a comprehensive diagnostic approach when multiple hematologic abnormalities coexist. Neither the plasma-cell clone nor the myeloid clone alone could account for the degree of gastrointestinal involvement, the dramatic elevation of alkaline phosphatase, or the burden of atypical mast cells. Similarly, mastocytosis alone would not explain the pattern of monocytosis or the presence of *TET2*/*SRSF2*/*RUNX1* mutations. Only the integration of all histologic, immunophenotypic, biochemical, and molecular data allowed for the correct categorization of the disease as SM-AHN with concurrent CMML-1 and t(11;14)+ plasma-cell neoplasm.

## 6 Conclusion

In summary, this case demonstrates how a seemingly nonspecific clinical presentation can ultimately reveal the coexistence of multiple clonal hematologic diseases, including advanced systemic mastocytosis, chronic myelomonocytic leukemia, and a t(11;14)-positive plasma-cell neoplasm. The diagnostic clarity achieved by integrating bone marrow morphology, immunophenotyping, cytogenetics, and mutational profiling was crucial in guiding an effective treatment strategy that simultaneously targeted the dominant mast-cell clone and the underlying myeloid disease. The early clinical improvement observed after therapy highlights both the importance of comprehensive evaluation and the value of individualized, mechanism-based treatment in patients with complex, overlapping hematologic disorders.

## Data Availability

The original contributions presented in the study are included in the article/**Supplementary Material**. Further inquiries can be directed to the corresponding author.
