# Supplementary material for: Case Report: Coexistence of advanced systemic mastocytosis, chronic myelomonocytic Leukemia, and t(11;14)-positive plasma-cell neoplasm: a rare triple-clonal hematologic case
Source: Front Oncol. 2026 Oct 19;16:1982665. doi: 10.3389/fonc.2026.1982665 (PMC13724441; doi:10.3389/fonc.2026.1982665)
Supplement: Supplementary file 1 [file Table1.docx]

**Supplementary Table 1** Timeline

| **Time point** | **Clinical events and findings** |
| --- | --- |
| 4 months before admission | Persistent watery diarrhea and recurrent abdominal discomfort developed. |
| Initial local-hospital evaluation | Chronic non-atrophic gastritis and duodenitis were identified. Laboratory tests showed absolute monocytosis of 1.54 × 10^9^/L and thrombocytopenia of 66 × 10^9^/L. |
| Admission to our hospital | Splenomegaly and bilateral lower-extremity edema were noted. Absolute monocyte count increased to 1.93 × 10^9^/L, hemoglobin was 100 g/L, platelet count was 62 × 10^9^/L, alkaline phosphatase was 1,145.3 U/L, and ferritin was > 1,500 ng/mL. |
| Diagnostic imaging and endoscopy | CT showed splenomegaly and mildly enlarged retroperitoneal lymph nodes. PET/CT showed intense gastric wall FDG uptake and increased splenic metabolism. Endoscopic biopsies showed chronic active gastritis and duodenal ulcers without specific infiltrative lesions. |
| Hematologic workup | Bone marrow examination showed 18.5% atypical mast cells in the aspirate and approximately 50% mast-cell infiltration in the biopsy. Flow cytometry identified aberrant mast cells and a lambda-restricted plasma-cell population. |
| Molecular and cytogenetic evaluation | KIT D816V, TET2, SRSF2, and RUNX1 mutations were detected. FISH identified t(11;14)(q13;q32)/IGH::CCND1. |
| Final diagnosis | Advanced systemic mastocytosis associated with CMML-1 and a t(11;14)-positive plasma-cell neoplasm. |
| Treatment | Avapritinib plus azacitidine was initiated. |
| After 3 cycles | Watery diarrhea decreased, hemoglobin and platelet counts improved, absolute monocyte count declined, and alkaline phosphatase showed a downward trend. |
